# Fever-Induced Brugada Syndrome

**DOI:** 10.1177/2324709615577414

**Published:** 2015-03-23

**Authors:** Sandhya Manohar, Binaya Raman Dahal, Bernard Gitler

**Affiliations:** 1 Montefiore New Rochelle Hospital, New Rochelle, NY, USA

**Keywords:** Brugada syndrome, fever, sudden cardiac death

## Abstract

Brugada syndrome is increasingly recognized as a cause of sudden cardiac death. Many of these patients do not get diagnosed due its dynamic and often hidden nature. We have come a long way in understanding the disease process, and its electrophysiology appears to be intimately linked with sodium channel mutations or disorders. The cardiac rhythm in these patients can deteriorate into fatal ventricular arrhythmias. This makes it important for the clinician to be aware of the conditions in which arrhythmogenicity of Brugada syndrome is revealed or even potentiated. We present such an instance where our patient’s Brugada syndrome was unmasked by fever.

## Introduction

Brugada syndrome is a cardiac arrhythmia syndrome that is often missed due to its dynamic nature. Its propensity to deteriorate into ventricular arrhythmias makes it a condition that, if missed, can be fatal. Understanding the circumstances that can reveal the hidden Brugada syndrome is crucial, not only for the practicing clinician but also for the patients so that they may be educated to promptly seek medical attention.

We present the case of a patient who presented with fever and was diagnosed with sepsis from a urinary tract infection but her electrocardiogram (ECG) taken while febrile demonstrated the Brugada ECG pattern that later reversed with defervescence.

## Case Report

A 74-year-old female came to our emergency department (ED) with fever and dysuria of 3 days duration. She was in Peru a week prior to presentation and had similar complaints. She was seen by a local physician there and was told that she had a urinary tract infection. She received a one-time injection of an unknown medication after which she felt much improved. She then travelled to the United States and had been here for 3 days after which her symptoms recurred.

Her medical history was notable for hypertension. She had no prior illness that required hospitalization and led a relatively healthy lifestyle. She had an unremarkable family history including no history of sudden death.

In the ED, she was noted to have a temporal temperature of 39°C, tachycardia at 101 bpm with a blood pressure of 129/49 mm Hg; her physical exam was unremarkable. Laboratory data were notable for leukocytosis with bandemia and pyuria. She was to be admitted for sepsis from a urinary tract infection. Within an hour she complained of increasing chills, and a repeat rectal temperature was 41°C. Prior to administration of antipyretics she developed a generalized tonic–clonic seizure. A computed tomography scan of the head was unremarkable. She was not on a cardiac telemetry at the time of the seizure.

**Figure 1.**
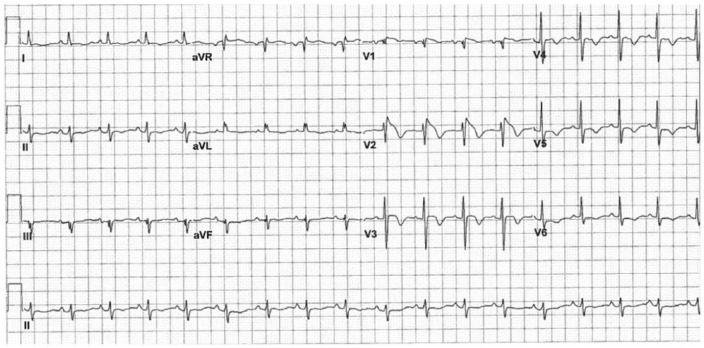
Initial electrocardiogram during a febrile episode showing type 1 Brugada pattern.

An ECG was obtained that showed sinus tachycardia at 106 bpm with coved ST segment elevation in leads V1 and V2 (more pronounced in V2), characteristic of the Brugada ECG pattern (Figure 1). Echocardiogram showed normal left ventricular systolic function with a left ventricular ejection fraction of 55% to 60% and no segmental wall motion abnormalities or valvular abnormalities. Her blood work was notable for mild elevation of troponin I at 1.5 ng/mL (normal reference range = 0.0-0.5 ng/mL), which was attributed to demand ischemia. Repeat ECGs during the febrile period continued to show the same precordial ST segment changes. Telemetry revealed no significant events. She had no further seizures. A neurologist was consulted who recommended no further workup for her first and one-time seizure episode.

**Figure 2.**
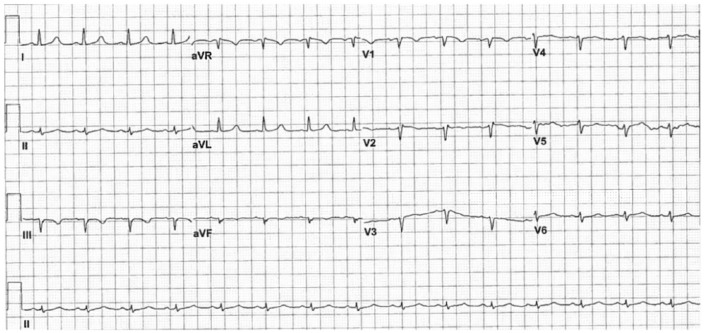
Electrocardiogram after defervescence showing resolution of the coved-type ST segment elevation in V1 and V2 leads.

After 48 hours of intravenous antibiotics, she showed clinical improvement and was no longer febrile. A repeat ECG with normal temperature demonstrated reversibility of her classic Brugada type 1 changes with clinical defervescence (Figure 2). She had no history of prior syncope, seizure, or palpitations. She refused to undergo any interventional studies; therefore, a cardiac catheterization and an electrophysiological study were not performed despite our recommendation.

At the time of discharge she was advised to have prompt intervention with antipyretics for any future febrile episodes and also to avoid certain medications (see Discussion). At 2-month follow-up, she remains well and free of cardiac events.

## Discussion

Brugada syndrome is a genetic disorder with a characteristic ECG finding of persistent or transient ST segment elevation in the right precordial leads (V1, V2) with or without right bundle branch block. These ECG changes are dynamic, often hidden, and may reveal themselves in the presence of triggers like fever, intoxication (alcohol, cocaine, or cannabis), vagal stimulation, electrolyte imbalance, anesthetics (propofol, bupivacaine), psychotropic agents (amitriptyline, lithium), and sodium channel blockers.^1^

Fever-induced Brugada syndrome is becoming a well-known entity. In a study by Amin et al,^2^ it was noted that fever was the precipitating factor for about 18% of the cardiac arrests in patients with symptomatic Brugada syndrome. Adler et al^3^ in their study found that a type I Brugada ECG pattern was 20 times more likely to occur in febrile patients than afebrile patients. This translated to a prevalence of 2%, whereas the estimated prevalence of asymptomatic Brugada syndrome in the general population is 0.05%.^4^ They also found the patients to be typically in an age group of 30 to 60 years, and 87% were of the male gender,^4^ a finding that was seen in other studies as well.^5^ This brings up an interesting possibility of an androgen hormone relationship to these patients with potential fatal ventricular arrhythmias.^4^ Though our patient is female, it is possible that her postmenopausal state contributed to the appearance of the Brugada ECG in this setting.

Two ECG patterns have been described in Brugada syndrome. Type 1 (coved pattern; which was seen in our patient) presents as ST-segment elevation of ≥2 mm (0.2 mV), which slowly descends followed by a symmetric negative T wave in the right precordial leads. There is no clear r^I^ wave. Type 2 (saddle-back pattern) begins with a positive wave called r^I^, which is ≥2 mm (0.2 mV) from the isoelectric line, followed by a minimum ST elevation ≥0.5 mm (0.05 mV) with a positive/flat T wave in V2 and a variable T wave in V1.^1^ The diagnosis of Brugada syndrome is mainly a clinical one. Along with the characteristic ECG pattern it requires one of the following clinical criteria: (*a*) a history of ventricular tachycardia (VT) or ventricular fibrillation (VF), (*b*) a family history of sudden cardiac death, (*c*) a family history of Brugada syndrome, (*d*) agonal respiration during sleep, or (*e*) inducibility of VT/VF during electrophysiological study.^4^

Several studies have noted that asymptomatic patients with Type 2 Brugada pattern have a benign outcome.^1,6^ On the other hand, patients with Type 1 Brugada pattern are almost 300 times more likely to have sudden cardiac death as compared with the general population.^7^ This is a risk regardless of whether the patient had spontaneous or induced Brugada pattern.^8^ This makes it useful to see if provocative ECG testing by intravenous administration of a sodium channel blocking drug (eg, flecainide, procainamide) turns a Type 2 pattern into a Type 1 pattern.^1,6^

Genetic analysis in patients with Brugada syndrome has shown an association with sodium channel *SCN5A* mutations in about 20% of the cases.^9^ This mutation results in an accelerated inactivation of sodium channels, which predisposes them to ventricular arrhythmias.^1^ Our patient had Brugada Type 1 ECG pattern induced by fever. Studies have noted that cardiac sodium channel mutations can result in temperature gated channels whose potential for arrhythmogenicity increases with higher temperatures.^10,11^ It can be hypothesized that our patient’s fever may have led to a self-terminating malignant arrhythmia causing relative cerebral hypoperfusion that led to her seizure. Prompt and aggressive control of fever is crucial in preventing malignant arrhythmias.^8^ Skinner et al^12^ report a case of a young child presenting similarly with febrile seizures who was subsequently diagnosed with Brugada syndrome, a diagnosis that was crucial in her case as it revealed a *SCN5A* mutation in her as well as her family members. A diagnosis of Brugada syndrome is important as these patients are at an increased risk for sudden cardiac death, despite a structurally normal heart.^13^

If our patient had an ECG done earlier as part of her routine workup in the ED, the diagnosis of Brugada could have alerted the physician for prompt and aggressive control of her fever. Although the US Preventive Services Task Force guidelines recommend against routine ECG screening for coronary heart disease in low-risk asymptomatic adult population, but there is no clear consensus on routine ECGs for patients being admitted to a hospital.^14^ As we discover more medications that affect the cardiac conduction system,^15^ it is maybe useful to have baseline ECGs on all patients.

Brugada syndrome has no known cure and prevention of malignant arrhythmias is the key principle guiding the therapy. This makes the treatment in asymptomatic patients with the Brugada ECG pattern controversial.^16^ Insertion of an implantable cardioverter-defibrillator (ICD) is the first line of therapy but the complications of invasive surgery and the negative psychological effects makes this a difficult therapeutic decision.^4^ The 2013 Heart Rhythm Society, European Heart Rhythm Association, and Asia Pacific Heart Rhythm Society guidelines support the use of an ICD in patients with a diagnosis of Brugada syndrome who also meet one of the following criteria: (*a*) patient has survived sudden cardiac death or has documented spontaneous sustained ventricular tachycardia or (*b*) patient has spontaneous type I Brugada ECG pattern with a history of syncope likely caused by a ventricular arrhythmia.^17^ Our patient did not meet the criteria for an ICD since it was unclear if she did have an undocumented malignant arrhythmia. An electrophysiological study is not routinely necessary in patients with asymptomatic Brugada ECG pattern but may have been beneficial in our case.

Drug therapy is mainly used for adjunctive therapy in patients having repeated ICD shocks. Quinidine and cilostazol have shown to help in VF suppression.^4^ Small studies have shown benefit of quinidine, but overall, drug therapy is not proven to be beneficial.^16,18^ Furthermore, quinidine is not a well-tolerated drug, and its side effects range from gastrointestinal issues to liver dysfunction and thrombocytopenia.^18^ Newer modalities of treatment including catheter ablation of epicardial substrate are still in experimental stages.^4^

In conclusion, making a diagnosis of Brugada syndrome can be difficult as the ECG patterns are often hidden. Being aware of the conditions in which they manifest are important for the practicing clinician. There are no clear-cut guidelines on the use of routine ECGs in patients admitted with noncardiac illnesses. We need to consider the effect of these disease processes on the cardiac conduction system and the effects of the medications that are given to treat them, to be able to better care for our patients.
